# Configurations of Splitter/Combiner Microstrip Sections Loaded with Stepped Impedance Resonators (SIRs) for Sensing Applications

**DOI:** 10.3390/s16122195

**Published:** 2016-12-20

**Authors:** Lijuan Su, Javier Mata-Contreras, Paris Vélez, Ferran Martín

**Affiliations:** CIMITEC, Departament d’Enginyeria Electrònica, Universitat Autònoma de Barcelona, Bellaterra, 08193 Barcelona, Spain; Lijuan.Su@uab.cat (L.S.); Franciscojavier.Mata@uab.cat (J.M.-C.); Paris.Velez@uab.cat (P.V.)

**Keywords:** stepped impedance resonator (SIR), microstrip technology, microwave sensors, differential sensors

## Abstract

In this paper, several configurations of splitter/combiner microstrip sections loaded with stepped impedance resonators (SIRs) are analyzed. Such structures are useful as sensors and comparators, and the main aim of the paper is to show that the proposed configurations are useful for the optimization of sensitivity and discrimination. Specifically, for comparison purposes, i.e., to determine anomalies, abnormalities or defects of a sample under test (SUT) in comparison to a reference sample, it is shown that up to three samples can be simultaneously tested. Simple models of the proposed structures are presented, and these models are validated through electromagnetic simulation and experiment. Finally, the principle of operation is validated through a proof-of-concept demonstrator.

## 1. Introduction

In recent years, there has been an increasing interest in microwave sensors. Many of these sensors are based on the variation of the resonance frequency, phase or quality factor of a resonant element, caused by the variable to be sensed [1,2,3,4,5,6,7,8,9]. Among them, there are sensors implemented by loading a transmission line with planar resonators coupled to it [8,9]. One limitation of these sensors is caused by the so-called cross sensitivity, i.e., the sensitivity of the sensing element to other variables. Particularly critical are the effects of changing environmental conditions, such as temperature or humidity, which may cause erroneous readouts and/or may force the sensing system to continuously calibrate the sensor in order to obtain reliable measurements. One possible solution to these limitations is differential sensing. The reason is that in differential sensors, the environmental factors are seen as common-mode stimulus. Therefore, their effects can be minimized and hence differential sensors are more robust in the face of variations in ambient factors.

Typically, differential sensors are based on two sensing elements, e.g., two transmission lines loaded with reactive elements or with resonators [10]. However, differential sensors can be also implemented by means of a single transmission line loaded with a pair of resonant elements [11,12], or with a pair of resonators coupled to it [13,14,15]. In this latter type of sensors, the sensing principle is based on frequency splitting. Namely, under a common-mode stimulus, e.g., identical dielectric loading in both resonators, a single transmission zero at the fundamental resonance frequency of the resonant elements arises. However, if the resonators are unequally loaded, two transmission zeros appear, and the distance between them is related to the difference in the loads.

It is also possible to implement sensors robust in the face of variations in ambient conditions by loading a transmission line with a single symmetric resonator. In this case, the sensing principle is based on symmetry properties, and specifically on the controllability of line-to-resonator coupling [16,17,18]. That is, by symmetrically loading a line with a symmetric resonator with its symmetry plane (aligned with the one of the line) being of different electromagnetic nature from the symmetry plane of the line (one an electric wall and the other one a magnetic wall), the resonator is not effectively coupled to the line. Under these conditions, resonance is prevented and the loaded line is transparent. However, if symmetry is truncated (e.g., by means of an asymmetric dielectric load, or by a relative displacement between the resonator and the line), the electric or magnetic field lines in the resonator area no longer cancelled, and line-to-resonator coupling appears, with the result of a frequency notch at resonance. Moreover, the notch depth, determined by the magnitude of coupling, is related to the level of asymmetry. Even though these sensors are not true differential sensors, they are scarcely affected by environmental factors, since such factors do not modify the symmetry conditions. Coupling-modulated resonance sensors have been applied to the implementation of linear and angular displacement sensors [16,19,20,21,22,23,24,25].

**Figure 1:**
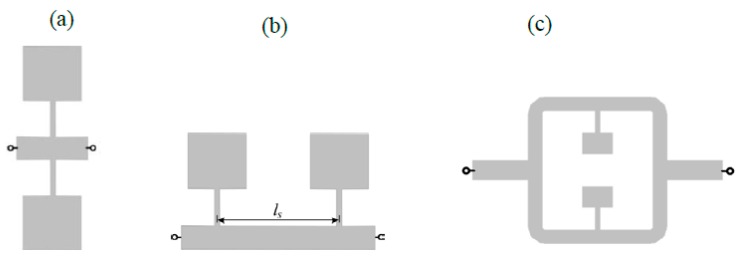
Various topologies of sensors based on microstrip lines loaded with SIRs. (**a**) Parallel configuration; (**b**) cascaded configuration; (**c**) splitter/combiner configuration.

This work is focused on frequency splitting sensors/comparators based on pairs of stepped impedance resonators (SIRs) [26,27] (other recently reported sensors based on pairs of resonant elements are reported in [28,29,30,31]). The first implementation of such sensors was presented in [11], where the sensing element consisted of a microstrip line loaded with a pair of SIRs placed at the same position in the line (parallel configuration, see Figure 1a). In this configuration, the SIRs are inductively coupled, and this causes sensitivity degradation at small perturbations, as discussed in [11,12]. One solution to prevent inter-resonator coupling is to place the SIRs at different positions along the line (cascade connection, see Figure 1b [12]). In this case, the transmission zeros are given by the intrinsic resonance frequencies of both resonators, and it was argued in [12] that by spacing the SIRs *λ*/2, where *λ* is the guided wavelength at SIR resonance, the equivalent circuit model is the one of the parallel configuration but without inter-resonator coupling.

In this paper, we discuss an alternative solution to avoid the coupling between SIRs: splitter/combiner microstrip sections, where each parallel line is loaded with a SIR (see Figure 1c). Nevertheless, various configurations for multi-sensing purposes, involving further number of SIRs and also based on parallel microstrip lines, are discussed in the paper.

## 2. Circuit Model and Analysis of SIR-Based Splitter/Combiner Sensors 

**Figure 2:**
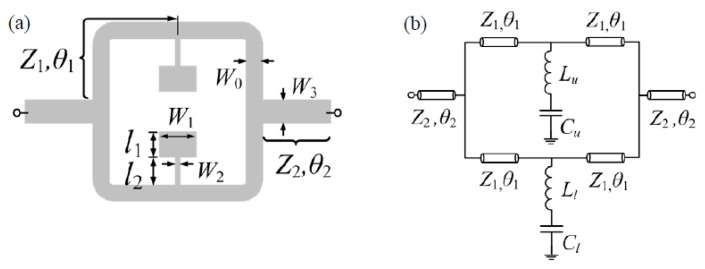
Topology and relevant dimensions of the SIR-loaded power splitter/combiner microstrip structure (**a**) and circuit schematic (**b**).

The typical topology (including relevant dimensions) of the considered SIR-loaded power splitter/combiner microstrip structure is depicted in Figure 2a. Each branch consists of a 50 Ω line loaded with a SIR. To match the structure to the 50 Ω ports, impedance inverters implemented by means of 35.35 Ω quarter wavelength transmission line sections are cascaded between the ports and the T-junctions. The circuit schematic, including distributed and lumped elements, is shown in Figure 2b, where the general case of an asymmetric structure is considered. However, asymmetry concerns SIR dimensions, rather than the distributed elements (transmission line sections). The lumped elements account for the SIRs; therefore, *L_u_*-*C_u_* and *L_l_*-*C_l_* model the upper and lower SIR, respectively. The distributed elements in the model describe the different transmission line sections, and are characterized by the line impedance *Z_i_* and electrical length *θ_i_* (with *i* = 1, 2).

It is assumed in the model that the SIRs are separated enough so as to neglect coupling between them. Nevertheless, note that unless the resonators are identical, situation that provides a single transmission zero at the fundamental SIR resonance, the two transmission zeros that should appear for asymmetric resonators (or unbalanced perturbations) are not given, in general, by the resonance frequencies of the SIRs. The reason is that in this splitter/combiner structure, a short in one of the parallel microstrip lines does not guarantee a transmission zero in the whole structure since the power can be transmitted through the other line. Indeed, for asymmetric SIRs, the transmission zeros are in general consequence of an interfering phenomenon. To gain insight on this, let us calculate the transmission zeros of the structure of Figure 2b. Such transmission zeros are given by the frequencies satisfying *Y*_21_ = *Y*_12_ = 0 [32], where *Y*_21_ = *Y*_12_ are the anti-diagonal elements of the admittance matrix (identical due to symmetry, with regard to the midplane between the ports, and reciprocity). Such element is given by:

(1) $$Y_{21}=Y_{21,u}+Y_{21,l}$$

where *Y*_21,*u*_ and *Y*_21,*l*_ are the anti-diagonal elements of the admittance matrix corresponding to the upper and lower SIR-loaded transmission lines. Such elements can be determined by first obtaining the *ABCD* matrix of each branch. It is simply given by the product of the matrices of the three cascaded two-port networks, including the two transmission line sections with impedance *Z*_1_, and the shunt connected series resonator (accounting for the SIR) in between. Once the *ABCD* matrices for each branch have been determined, the elements of the right-hand side in Equation (1) are given by *Y*_21,*u*_ = −1/*B_u_* and *Y*_21,*l*_ = −1/*B_l_*, where *B_u_* and *B_l_* are the *B* elements of the *ABCD* matrix for the upper and lower parallel branches, respectively [32]. Thus, the transmission zeros are given by:

(2) $$\frac{1}{B_{u}}+\frac{1}{B_{l}}=0$$

with:

(3a) $$B_{u}=jZ_{1}\;\text{sin}\;2\theta_{1}-\frac{Z_{1}^{2}\;{\text{sin}}^{2}\;\theta_{1}}{Z_{u}}$$

(3b) $$B_{l}=jZ_{1}\;\text{sin}\;2\theta_{1}-\frac{Z_{1}^{2}\;{\text{sin}}^{2}\;\theta_{1}}{Z_{l}}$$

*Z_u_* and *Z_l_* being the impedance of the SIR of the upper and lower branch, respectively. i.e.,:

(4a) $$Z_{u}=j\left(\omega L_{u}-\frac{1}{\omega C_{u}}\right)$$

(4b) $$Z_{l}=j\left(\omega L_{l}-\frac{1}{\omega C_{l}}\right)$$

Inspection of Equations (2) and (3) reveals that if either *Z_u_* or *Z_l_* are zero (both cannot be zero simultaneously unless the resonance frequencies of both resonators are identical), a transmission zero does not arise. If the resonators are different and exhibit different resonance frequencies, the transmission zeros appear at those frequencies where *B_u_* = −*B_l_*, and are consequence of signal interference. There is, however, a particular situation not accounted for by Equation (2). It corresponds to the case where the distance between the T-junction and one of the SIRs is *λ*/2, or *θ*_1_ = *π*. In this case, one of the transmission zeros is given by the resonance frequency of the corresponding SIR, whereas the other one is given by the solution of Equation (2). Note that if *θ*_1_ = *π*, the shunt reactance is translated to the T-junction, providing a short at this point and hence a transmission zero, regardless of the characteristics of the other parallel branch. In this case, Y11 = ∞ and Y21 ≠ ∞.

## 3. Model Validation and Sensitivity Optimization

**Figure 3:**
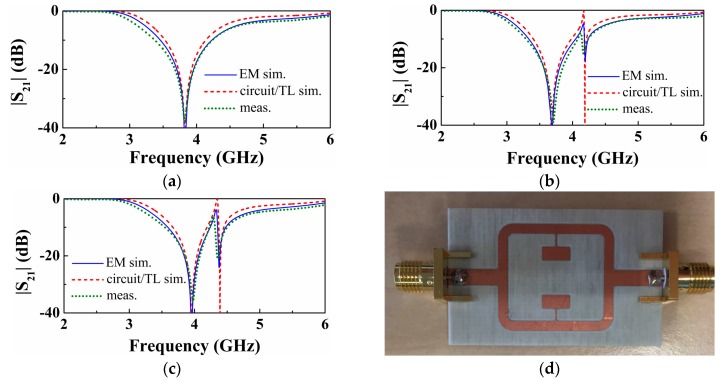
Insertion losses for the structure of Figure 2. (**a**) For the symmetric structure with both SIRs identical to the upper SIR of Figure 2, with *l*_1,*u*_ = *l*_1,*l*_ = 2.6 mm; (**b**) for the asymmetric structure with lower SIR having bigger capacitance (increasing 0.494 mm or Δ*l*_1,*l*_ = 0.19*l*_1,*l*_) and (**c**) for the asymmetric structure with lower SIR having smaller capacitance (decreasing 0.494 mm or Δ*l*_1,*l*_ = – 0.19*l*_1,*l*_). For all three cases, the dimensions of the others are the same: *W*_1,*u*_ = *W*_1,*l*_ = 5.5 mm, *W*_2,*u*_ = *W*_2,*l*_ = 0.25 mm, *l*_2,*u*_ = *l*_2,*l*_ = 2.6 mm, *W*_0_ = 1.84 mm, *W*_3_ = 3.1 mm, *θ*_1_ = 0.8π, *θ*_2_ = 0.5π. The reactive parameters are: (**a**), *L_u_* = *L_l_* = 2.59 nH, *C_u_* = *C_l_* = 0.67 pF; for (**b**), *C_l_* = 0.77 pF and for (**c**), *C_l_* = 0.56 pF, with the other parameters the same as in (**a**). The photograph of the fabricated structure corresponding to the symmetric case is depicted in (**d**).

For the validation of the model of the SIR-based structures under study, let us consider the topology of Figure 2 with the geometric parameters indicated in the caption of Figure 3, and the RO4003C substrate (Rogers, CT, USA) with thickness *h* = 812.8 μm and dielectric constant ε_*r*_ = 3.38. The insertion losses inferred from electromagnetic simulation (using the Momentum software, Keysight, Santa Rosa, CA, USA) are depicted in Figure 3a.

The reactive parameters of the two resonators can be easily inferred from independent simulations of single SIR-loaded transmission lines. From the resonance frequency (transmission zero) and reactance slope, *L_u_*, *C_u_* and *L_l_*, *C_l_* can be obtained. The circuit response obtained from the extracted element values (indicated in the caption of Figure 3), is also depicted in Figure 3a, where the good agreement with the electromagnetic response can be appreciated. Finally, we have fabricated the structure of Figure 2, and we have experimentally obtained the insertion losses, also included in Figure 3a, by means of the N5221 vector network analyzer (Agilent, Santa Clara, CA, USA). Again, the agreement with the electromagnetic and circuit response is reasonable (small discrepancies are due to fabrication related tolerances and to some uncertainties in the nominal value of the dielectric constant of the considered substrate).

We have repeated the previous procedure by varying the dimensions of one of the SIRs (particularly the dimensions of the capacitive patch of the lower SIR). Specifically, we have considered asymmetric structures with the patch capacitances of the lower SIR smaller or larger than the one of the upper SIR (contrary to the symmetric case described in the previous paragraph). The frequency responses of these two asymmetric cases are depicted in Figure 3b,c, and the agreement between the simulated and experimental responses is good as well. Therefore, with these results, corresponding to different situations, the model is validated.

**Figure 4:**
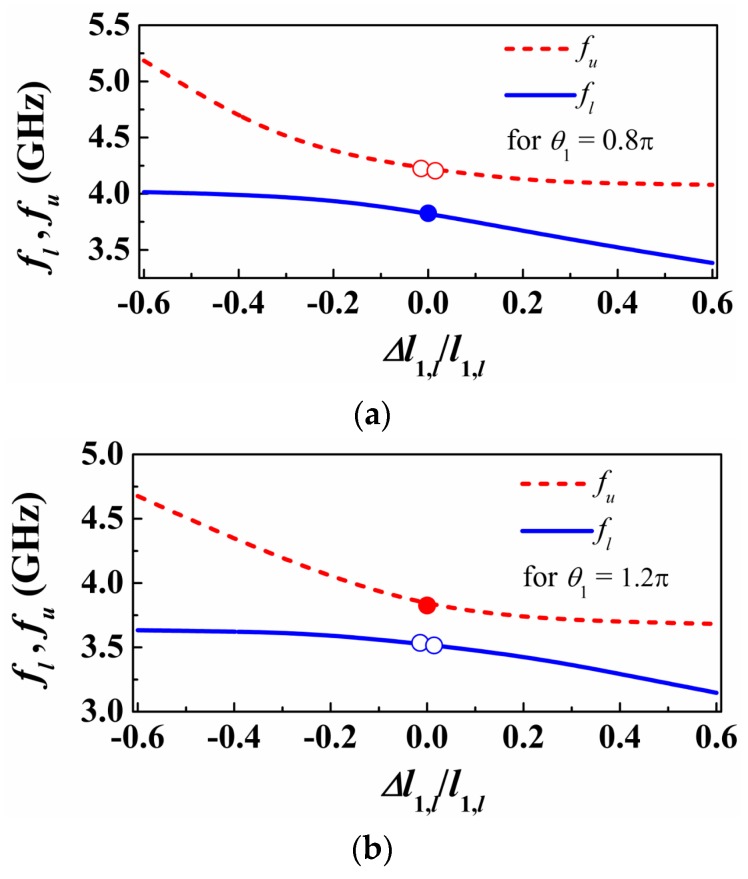

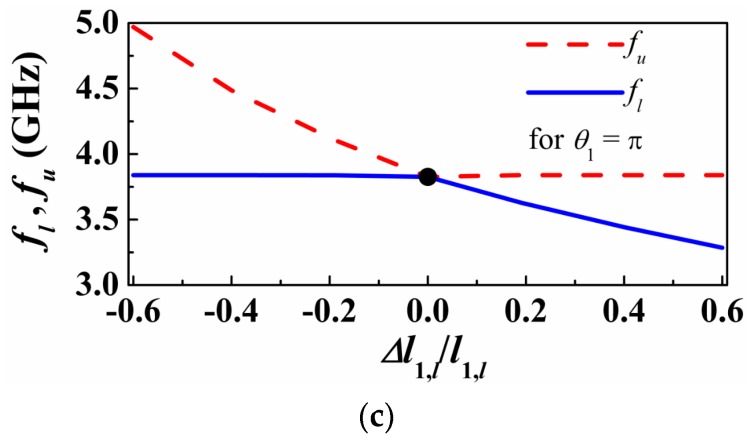
Variation of the transmission zeros as a function of the length of one of the SIR capacitive patch, for different electrical length of the transmission lines: (**a**) θ_1_ = 0.8π, (**b**) θ_1_ = 1.2π, (**c**) θ_1_ = π.

We have considered further asymmetric structures resulting by further increasing or decreasing the length of the patch capacitance of the lower SIR. In all the cases, we have obtained the transmission zeros as a function of this length (Figure 4a). As can be seen, the transmission zeros tend to separate as the asymmetry increases. However, such zeros do not cross in the limit of small asymmetries (emulating small asymmetric perturbations or loads). Hence, the sensitivity, defined as the variation of the differential output with the differential input, is degraded in this limit. Note that the differential output is the difference between the transmission zeros, whereas the differential input can be, for instance, the difference in the capacitances of the SIRs. This phenomenology is also present in structures consisting of single transmission lines loaded with parallel SIRs [11].

In the structures corresponding to Figure 4a, the electrical length of the lines satisfies θ_1_ < π, specifically θ_1_ = 0.8π. Let us now repeat the previous procedure by considering θ_1_ = 1.2π > π. The resonance frequencies are depicted in Figure 4b. A similar phenomenology arises, but now the single resonance frequency corresponding to the symmetric structure appears on the opposite curve. Finally, let us consider the situation with θ_1_ = π. In this case, the curves giving the two transmission zeros cross (Figure 4c), and one of the transmission zeros does not vary with the level of asymmetry, as anticipated before. This latter case is the preferred one in terms of sensitivity optimization. By choosing θ_1_ = π we achieve a similar performance as the one of the cascaded configuration [12].

## 4. Multi-Sensing Structure

**Figure 5:**
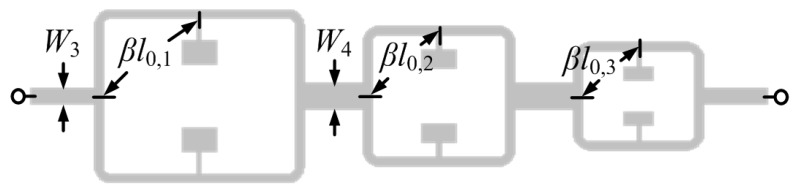
Splitter/combiner sections loaded with three pairs of SIRs for multi-sensing purposes. Dimensions are: *W*_3_ = 3.1 mm (35.35 Ω line), providing impedance matching to the 50 Ω input and output ports; *W*_4_ = 4.9 mm, for connecting the middle subsection, providing a 25 Ω line for good impedance matching. The lengths of the lines connecting the splitter/combiner sections and the lines adjacent to the ports are 12.1901 mm and 12.4495 mm, given at the center frequency of the working frequency range (3.6 GHz). For each sub-section, *l*_0,1_ = 32.2192 mm, given at intrinsic resonant frequency of the first sub-section’s SIR( 2.852 GHz) with electric length of π; *l*_0,2_ = 23.9817 mm, given at intrinsic resonant frequency of the middle sub-section’s SIR (3.826) GHz with electric length of π; *l*_0,3_ = 19.0571 mm, given at intrinsic resonant frequency of the third sub-section’s SIR (4.807 GHz) with electric length of π. SIR dimensions are: the first section with *W*_1_ = 5.5 mm, *l*_1_ = 3.5 mm, *W*_2_ = 0.25 mm, *l*_2_ = 3.8 mm; the middle sub-section with *W*_1_ = 5.5 mm, *l*_1_ = 2.6 mm, *W*_2_ = 0.25 mm, *l*_2_ = 2.6 mm; the third sub-section with *W*_1_ = 4.5 mm, *l*_1_ = 1.8 mm, *W*_2_ = 0.25 mm, *l*_2_ = 2.6 mm (each sub-section is symmetric, so the SIRs have the same dimension as mentioned above). The substrate is identical to the one of Figure 2.

**Figure 6:**
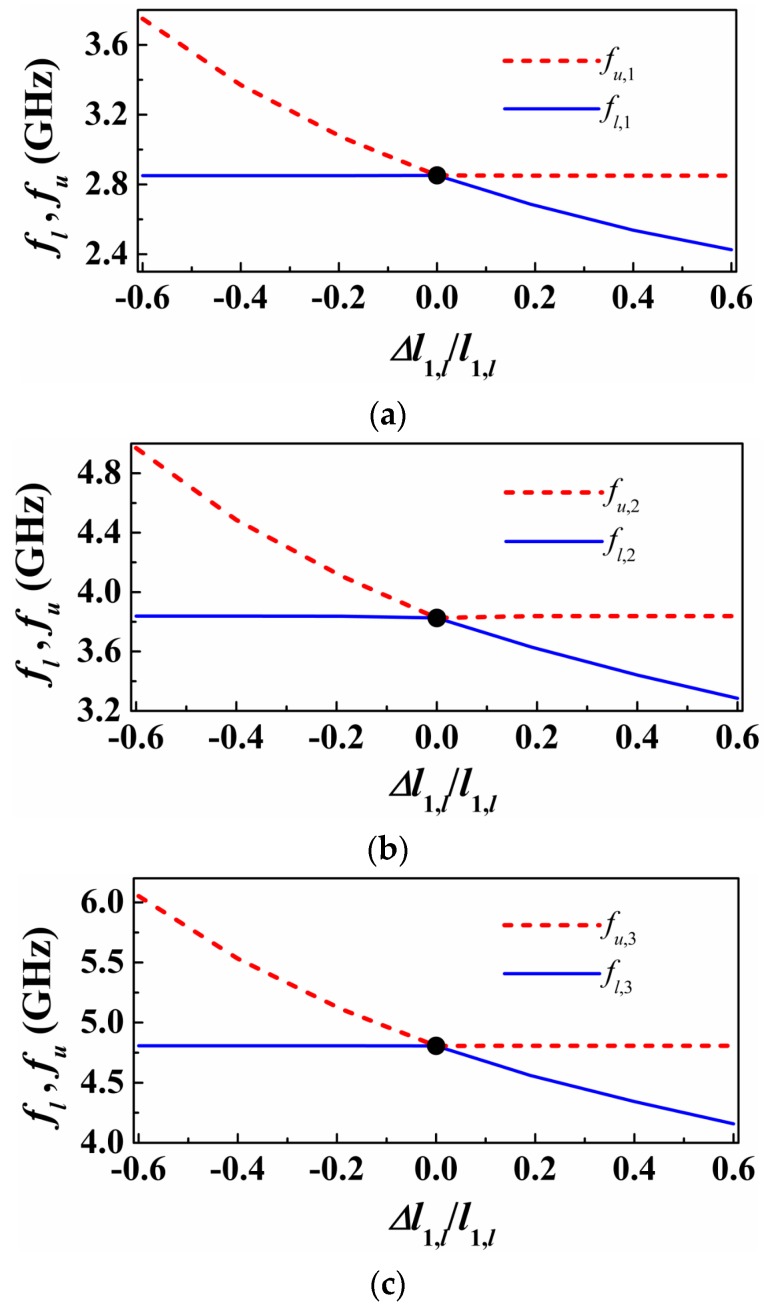
Variation of the transmission zeros as a function of the length of one of the SIR capacitive patch. (**a**) the first sub-section; (**b**) the middle sub-section; (**c**) the third sub-section. For each figure, only one pair of SIRs is unbalanced (*f_u,i_* and *f_l,i_* – *i* corresponding for three different sub-sections).

**Figure 7:**
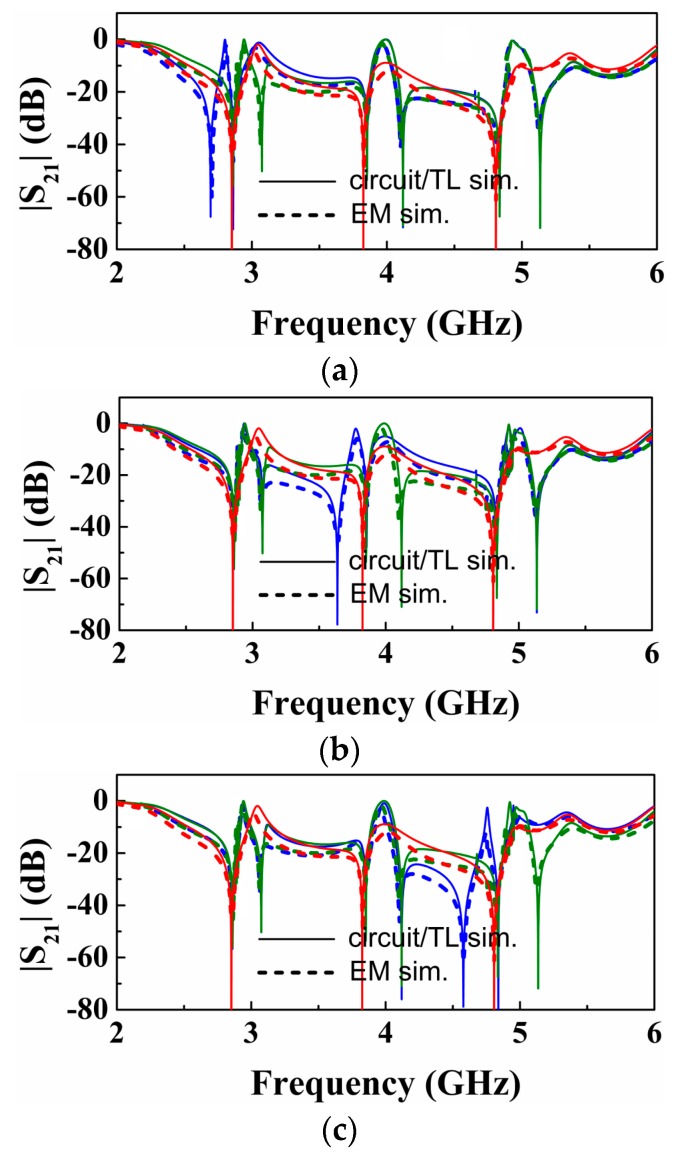
Magnitude of the transmission coefficient obtained from lossless electromagnetic and circuit simulations. In all three cases, red curve is for symmetric case, where blue and olive curves correspond to asymmetric cases for different sections with bigger and small SIRs, respectively. (**a**) the capacitor patch length of the lower SIR for the first sub-section increased/decreased 0.665 mm (Δ *l*_1,*l*_ = ±0.19, *l*_1,*l*_ = ±0.665 mm), while the second and third sub-sections being asymmetric by decreasing the length of the lower capacitive patch 0.19 *l*_1,*l*_ for each SIR patch length individually; (**b**) the capacitor patch length of the lower SIR for the second sub-section increased/decreased 0.494 mm (Δ *l*_1,*l*_ = ±0.19, *l*_1,*l*_ = ±0.494 mm), while the first and third sub-sections being asymmetric by decreasing the length of the lower capacitive patch 0.19 *l*_1,*l*_ for each SIR patch length individually; (**c**) the capacitor patch length of the lower SIR for the third sub-section increased/decreased 0.342 mm (Δ *l*_1,*l*_ = ±0.19, *l*_1,*l*_ = ±0.342 mm), while the first and second sub-sections being asymmetric by decreasing the length for the lower capacitive patch 0.19 *l*_1,*l*_ of each SIR patch length individually.

The structure of Figure 2 can be useful as a sensor for permittivity measurement or as comparator, where a single sample (sample under test -SUT) can be tested. In order to simultaneously characterize multiple samples, several splitter/combiner sections like the one shown in Figure 2, each one loaded with a pair of SIRs tuned at different frequencies, can be cascaded (Figure 5). With this configuration, in absence of loading or with balanced loads in each pair of SIRs, three transmission zeros (each one at the resonance frequency of the SIRs) are expected. However, with an unbalanced perturbation in a pair of SIRs, it is expected that the corresponding frequency splits. By the placement of the pairs of SIRs at a distance of λ/2 from the T-junctions, sensitivity and discrimination for small unbalanced perturbations is optimized, as discussed before. This is corroborated in Figure 6, where the split frequencies for each pair of SIRs, achieved by varying the patch dimensions of one of the SIRs of the pair, are depicted. As can be seen, the transmission zero frequencies cross, this being indicative of sensitivity and discrimination optimization. In Figure 6, only one pair of SIRs is unbalanced (the others are kept unaltered). However, by inferring the split frequencies (transmission zeros) by considering the other two pairs unbalanced as well, similar results are obtained, as corroborated in Figure 7.

## 5. Proof-of-Concept Demonstrator

**Figure 8:**
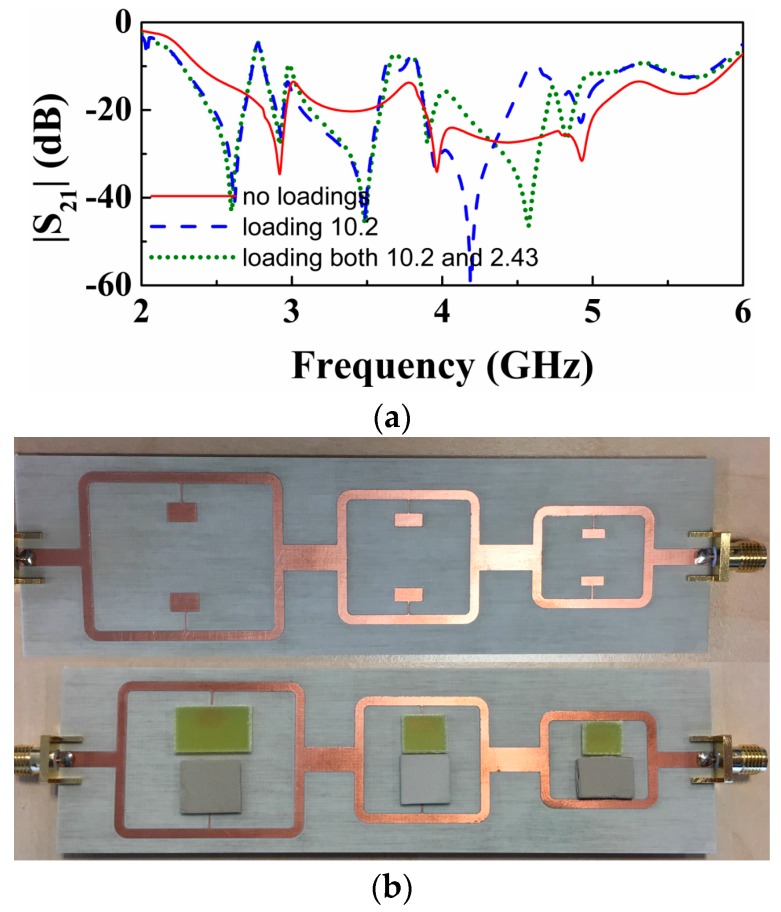
(**a**) Measured response (insertion loss) of the structure of Figure 5, compared with the response that results by loading one of the SIRs of every pair with a dielectric slab of dielectric constant 10.2, and by loading both SIRs of each pair with dielectric slabs of different dielectric constant (10.2 and 2.43); (**b**) The photograph of the fabricated structure corresponding to the symmetric case and when it is loaded with two different dielectric slabs for each pair of every section. The considered dimensions of the slabs are: for un-metalized *Rogers RO3010* (the bottom three loading slabs in Figure 2b), the average dimension is around 10 mm × 10 mm; and for Arlon CuClad 250 LX (the upper three loading pieces of slabs in Figure 2b), the biggest slab with 15.5 mm × 9.6 mm, the middle slab with 10 mm × 10 mm and the smallest slab with 9.3 mm × 8.0 mm, which is obvious that all slabs are much more bigger than the SIRs.

We have fabricated the structure of Figure 5. The measured response, depicted in Figure 8, exhibits three transmission zeros, as anticipated before. To demonstrate the potential of this structure as multi-sensor based on frequency splitting, we have asymmetrically loaded the three pairs of SIRs. To this end, we have simply added a dielectric slab (a square-shaped piece of un-metalized Rogers RO3010 substrate with thickness *h* = 1.27 mm and dielectric constant ε_*r*_ = 10.2) on top of one of the SIRs of each pair. With these unbalanced loads, frequency splitting of the three resonance frequencies of the SIRs is expected, and this is confirmed from the measured response, also included in Figure 8. Note that one of the notches for each split resonance coincides with the one of the symmetric structure. The reason is that asymmetry has been achieved by keeping one of the SIRs of each pair unaltered (i.e., without dielectric on top of it).

Then we have considered another experiment, where each pair of SIRs has been loaded with unbalanced loads, but in this case considering dielectric slabs with different dielectric constants (i.e., square-shaped pieces of un-metalized substrate with thickness *h* = 1.27 mm and dielectric constants ε_r_ = 10.2 and Arlon CuClad 250 LX with ε_r_ = 2.43 and *h* = 0.49 mm).

Again, frequency splitting (see Figure 8) points out the difference in the dielectric constants of both slabs loading the different pairs of SIRs. In this case, however, the two notches are shifted to lower frequencies, as compared to the resonance positions for the symmetric case. It is interesting to highlight that the notch positions for the structure with two different slabs for each pair are a result of an interfering phenomenon. For that reason the pair of notches generated by each splitter/combiner section does not coincide with those for the previous case.

Note that the experiment of Figure 8 is simply a proof of concept to demonstrate the possibility of performing multi-sensing measurements simultaneously. The transmission zeros are in general dependent on the size and thickness of the samples, but if size and thickness are high enough, so that the electromagnetic field in the SIR region (patches) is within the sample and substrate, then the transmission zeros depend only on the dielectric constant of the material. This means that in order to carry out accurate measurements this condition is necessary. In our case, the considered samples have an area much larger than the patches of the SIRs. The thickness may not be sufficient to guarantee the previous condition, but, as mentioned, our aim was simply to demonstrate the possibilities of the approach for multi-sensing. Note, however, that for comparison purposes, i.e., to evaluate if two apparently identical samples have differences, the thickness of the samples could be small if it is identical in both samples. It can be appreciated in Figure 8 that the samples in each pair are tiny separated; however, the SIRs are separated enough so that coupling between them can be discarded.

## 6. Conclusions

In conclusion, sensing structures based on splitter/combiner microstrip sections loaded with pairs of stepped impedance resonators (SIRs) have been studied in detail. First of all, we have considered a splitter/combiner loaded with a pair of SIRs situated in the mid-plane between the input and the output port. We have proposed a circuit schematic of the structure, which has been validated by comparison of the circuit response with extracted parameters with electromagnetic and measured responses, where both symmetric and asymmetric configurations have been considered. It has been shown that by placing the SIRs at a distance of half wavelength from the T-junctions of the splitter/combiner section, the sensitivity and discrimination of the structure as sensor or comparator is optimized. Finally, a configuration, based on three splitter/combiner sections, able to simultaneously sense or compare three samples, has been proposed. We have also demonstrated the potential of this multi-sensing structure by asymmetrically loading the pairs of SIRs and measuring the frequency response, where frequency splitting reveals the presence of unbalanced loads.
